# The Roles of Polysaccharides in Carp Farming: A Review

**DOI:** 10.3390/ani13020244

**Published:** 2023-01-10

**Authors:** Khang Wen Goh, Zulhisyam Abdul Kari, Wendy Wee, Hien Van Doan, Mohd Farhan Hanif Reduan, Muhammad Anamul Kabir, Martina Irwan Khoo, Syed M. Al-Amsyar, Lee Seong Wei

**Affiliations:** 1 Faculty of Data Science and Information Technology, INTI International University, Nilai 71800, Malaysia; 2 Department of Agricultural Sciences, Faculty of Agro-Based Industry, Universiti Malaysia Kelantan, Jeli Campus, Jeli 17600, Malaysia; 3 Advanced Livestock and Aquaculture Research Group, Faculty of Agro-Based Industry, Universiti Malaysia Kelantan, Jeli Campus, Jeli 17600, Malaysia; 4 Center of Fundamental and Continuing Education, Universiti Malaysia Terengganu, Kuala Nerus 21030, Malaysia; 5 Department of Animal and Aquatic Sciences, Faculty of Agriculture, Chiang Mai University, Chiang Mai 50200, Thailand; 6 Science and Technology Research Institute, Chiang Mai University, 239 Huay Keaw Rd., Suthep, Muang, Chiang Mai 50200, Thailand; 7 Department of Paraclinical Study, Faculty of Veterinary Medicine, Universiti Malaysia Kelantan, Pengkalan Chepa, Kota Bharu 16100, Malaysia; 8 Department of Aquaculture, Sylhet Agricultural University, Sylhet 3100, Bangladesh; 9 Department of Chemical Pathology, School of Medical Sciences, Universiti Sains Malaysia, Health Campus, Kubang Kerian 16150, Malaysia; 10 Faculty of Agro-Based Industry, Universiti Malaysia Kelantan, Jeli Campus, Jeli 17600, Malaysia

**Keywords:** growth performance, immune system, disease resistance, abiotic stressor, synergistic, sustainable aquaculture

## Abstract

**Simple Summary:**

Disease outbreaks pose a major challenge for farmers in sustaining carp farming. Antibiotics and chemicals are the conventional solutions to improve carp growth and health performance. Nonetheless, the consistent use of antibiotics as an antimicrobial agent in aquaculture could lead to pathogen resistance, harm the beneficial gut microorganisms and environment, and cause the build-up of excess antibiotics in the fish muscle that is dangerous to humans. Alternatively, polysaccharides could improve growth performances, active innate immunity and disease resistance, and alleviate abiotic stress in carp and other aquatic species. The application of polysaccharides in carp farming varies depending on the dosage and combination with other agents. Therefore, a comprehensive study is essential to reveal the molecular and cellular pathways that regulate the immune responses of carp to different pathogens and abiotic stressors.

**Abstract:**

Carp is an important aquaculture species globally, and the production is expected to increase with the growing market demands. Despite that, disease outbreaks remain a major challenge, impeding the development of sustainable carp farming. Moreover, the application of antibiotics, a common prophylactic agent, can adversely impact public health and the environment. Therefore, polysaccharide has been recognized as a novel prophylactic agent in the health management of carp farming, as well as gaining consumers’ confidence in carp farming products. In this review, the definition, sources, and main roles of polysaccharides in improving growth performance, stimulating the immune system, enhancing disease resistance, and alleviating abiotic stresses in carp farming are discussed and summarized. In addition, the use of polysaccharides in combination with other prophylactic agents to improve carp farming production is also highlighted. This review aims to highlight the roles of polysaccharides and provide valuable information on the benefits of polysaccharides in carp farming.

## 1. Introduction

Aquaculture contributed to approximately 46% of world fish production in 2018 [1]. More than half of the aquaculture production consisted of tilapia and carp farming [2], with significant production in India (16.10%) and Bangladesh (4.07%). In China, approximately 5 million tons of grass carp production alone was recorded in 2017. World carp production was recorded at nearly 28,000 thousand tons and contributed approximately 56.2% of the total world aquaculture production [1]. Various commercial carp species, such as grass carp, silver carp, common carp, bighead carp, catla, and rohu, are farmed for food worldwide, while goldfish and koi are reared as ornamental fish [2]. Furthermore, carp production is expected to increase with the expansion of the carp farming system and rising market demand.

Diseases threaten the sustainable development of carp farming and other aquaculture species, thus leading to significant economic losses [3]. Pathogens, such as parasites, fungi, bacteria, and viruses, have been reported to infect carp. Bacterial diseases caused by *Aeromonas hydrophila*, *Flexibacter columnaris*, and *Citrobacter freundii* are common in carp [4], while *Yersinia ruckeri* was recently reported to cause an enteric red mouth disease outbreak in Indian major carps [5]. Viral diseases, such as those caused by *Rhabdovirus carpio* [6] and koi herpesvirus disease (KHVD), have devastated numerous carp farms. Sunarto et al. [7] reported that KHVD wiped out many koi farms within three months in East Java, Indonesia, recording a total loss of USD 500 million. Recently, a viral disease outbreak by carp edema virus (CEV) was claimed to cause mass fish mortality in koi farms in China [8]. Moreover, abiotic factors such as low pH, low oxygen level, and the presence of pesticides and heavy metals in the water were also associated with mortalities in carp farming. Various studies have been conducted to enhance aquatic species’ growth and health performances. Traditionally, antibiotics and chemicals were the temporary solutions to curb disease outbreaks in aquaculture. The abuse of these substances and antibiotics in carp farming eventually led to the rise in antibiotic resistance among pathogenic bacteria and the exposure of carps to high levels of antibiotic and chemical residues, posing a threat to public health and the environment. Alternatively, prophylactic agents such as polysaccharide are potentially useful for health management in carp farming and boosting farm production. In this review, the application of polysaccharides in carp farming to improve growth, enhance immune system, stimulate disease resistance, and mitigate abiotic stress factors is discussed and summarized.

## 2. Polysaccharide

A polysaccharide is a simple sugar polymer consisting of monosaccharides linked together with glycosidic linkages [9]. Homopolysaccharide comprises the same monosaccharide, while heteropolysaccharide consists of different monosaccharides. The molecular structure of polysaccharides can be linear or highly branched [9]. Polysaccharides can be sourced from microorganisms, plant cell walls, plant seeds, medicinal herbs, seaweeds, and agricultural wastes [10]. For instance, β-glucan is a well-known polysaccharide that can enhance and sustain hosts’ health, as well as promoting the growth of beneficial microbiota in their gut. Another valuable polysaccharide to maintain a host’s health is pectin [11], which is derived from fruits and plants. On the other hand, fucoidan is derived from seaweed [12]. Pectin and fucoidan possess antimicrobial properties that can inhibit the growth of various bacteria and viruses. In summary, the polysaccharide is a bioactive compound derived from multiple sources and utilized as a functional food additive to maintain host health.

Chitin, pectin, starch, hemicellulose, and inulin are polysaccharides that are reportedly used in carp farming to boost production. Chitin is made of a glucose derivative, N-acetylglucosamine, derived from aquatic animals such as crab and shrimp shells. Furthermore, chitin is found in insect exoskeletons. Chitin deacetylation produces chitosan, an important chitin derivative [13]. Chitosan is soluble in acid and widely used in pharmaceuticals, food, and cosmetics. Previous studies reported that chitosan could stimulate plant growth and regulate various fungi, viruses, and bacteria [14]. Pectin is found in plants, comprising methylated polygalacturonic acid units with a carboxylic group. This mucopolysaccharide is a good source of fiber and cholesterol, possesses medicinal properties for blood sugar and insulin regulation, and helps in drug distribution throughout the human body [15]. Pectin cannot be absorbed, but it is an excellent binder for microorganisms, toxins, and harmful substances in the digestive system. Additionally, pectin can regulate the pH level in the host’s digestive system [16].

Hemicellulose can be found in the middle lamella of plant cell walls, thus making it a crucial component of the plant cell. This polysaccharide consists of pentose (C5) (arabinose and xylose) and hexose (C6) (galactose, glucose, and mannose) sugars linked together [17]. Furthermore, hemicellulose could enhance the immune system and exhibits anticancer properties [17]. Starches such as wheat bran polysaccharide and β-glucan are also used in carp farming. Starch is a polysaccharide made from glucose monomers linked together with α 1, 4 linkages and is an essential energy provider and immune system booster. Meanwhile, inulin can also supply energy, promote probiotic growth (*Bifidobacterium* and *Lactobacilli)*, and help absorb minerals required by the immune system [18]. In summary, polysaccharide properties and modes of action benefit host health. Generally, polysaccharides can control the food transit time in the digestive system by increasing bulkiness in the intestinal tract, influencing the digestion and absorption rate, and promoting short-chain fatty acids (SCFAs) production in the colon [19]. Therefore, polysaccharides can be used as feed additives to enhance animal health and growth.

## 3. Role of Polysaccharides and Growth Performance Improvement in Carp Farming

Most polysaccharides that are used to improve carp growth performance originate from medicinal herbs, seaweeds, and agricultural wastes. In addition, commercial polysaccharides such as allicin, rare earth–chitosan chelate, and xylooligosaccharides (XOS) are employed to boost carp farming growth performance. Polysaccharides derived from medicinal herbs such as allicin [20], *Lycium barbarum* [21], *Astragalus membranaceus* [22], *Ficus carica* [23], *Astragalus* spp. [24], and *Taraxacum mongolicum* [25] have been claimed to help improve carp farming performance (Table 1). The modes of action of the medicinal herb-derived polysaccharides in promoting fish growth have been extensively studied, specifically the secondary metabolites, to determine the biological activities of the herbs [26].

Secondary metabolites in medicinal plants help promote fish growth and serve as prebiotics for fish gut microbiota [27]. Consequently, feed digestibility increases and contributes to the fish growth performance. According to Awad and Awaad [28], the protein in seeds of medicinal herbs could promote fish growth. Furthermore, black cumin (*Nigella sativa*) seed cake at a certain percentage was proven to be a suitable protein replacement in the feed formulation of mirror carp fingerlings, while a high level of this ingredient adversely affected the growth performance of mirror carp [29]. Therefore, polysaccharides from medicinal herbs should be included at an optimum level to impact carp growth performance positively. Moreover, bioactive compounds from seaweeds such as the *Porphyra yezoensis* polysaccharide [30], fucoidan derivative from *Undaria pinnatifida* [31], *Enteromorpha prolifera* polysaccharide [32], and alginate oligosaccharide [33] are significant in promoting carp growth performance [34] (Table 1). Vidhya Hindu et al. [35] claimed that seaweed polysaccharides as prebiotics promoted the establishment of healthy gut microbiota and enhanced fish feed digestion. Furthermore, Cui et al. [31] reported that fucoidan simultaneously promoted gut microbiota growth and gibel carp (*Carassius auratus gibelio*).

Agricultural waste management has become a constant problem in food production; thus, using agricultural waste for polysaccharide production helps sustain the agriculture industry [36]. Various efforts have been conducted to fully utilize agricultural waste, reducing the environmental burden [37]. For instance, polysaccharides derived from agricultural wastes, such as fermented wheat bran polysaccharide [4] and pectin derived from orange peel [38] and apple peel [39], boosted the carp growth performance (Table 1). Meanwhile, pectin is a fruit fiber that promotes the growth of lactic acid bacteria, the most common type of probiotic [40] that can increase host feed digestibility, thus improving the feed conversion rate and lower feed cost. Recent findings demonstrated that utilizing wheat bran and fruit peel in carp farming could be beneficial, leading to sustainable carp farming.

Multiple studies have claimed that polysaccharides are beneficial for carp farming, but when in excess, polysaccharide poses a threat to carp growth. For example, overdosing common carp with galactomannan-rich sesbania (*Sesbania aculeata*) seed resulted in poor growth performance [41]. Meanwhile, chitosan at >10,000 mg/kg diet suppresses gibel carp’s growth performance [42]. Conversely, feed utilization and growth performance of gibel carp were not negatively impacted when guar gum was supplemented at a dose of 1% of the diet. Nevertheless, their growth performance was affected due to the decreased feed intake and digestive enzyme activities when guar gum was supplemented at a dose of 5% of the diet [43]. In conclusion, polysaccharides from agricultural wastes help increase growth performance in carp farming when administered at an appropriate dose.

**Table 1:** Polysaccharides in improving growth performance of carp farming.

Polysaccharides (Category)	Species and Weight	Dose	Duration/Degree of Growth Improvement	Reference
Rare earth–chitosan chelate (Chitin)	Gibel carp, *Carassius auratus gibelio*; 14.3 g	0.8 g/kg diet	12 weeks 12–20% higher than control	[44]
*Porphyra yezoensis* polysaccharide (Pectin)	Grass carp, *Ctenopharyngodon Idella*; 6.12 g	3 g/kg diet	60 days >8% higher than control	[30]
*Radix rehmanniae preparata* polysaccharide (Inulin)	*Luciobarbus capito*; 46 g	0.05–0.4% of diet	60 days 2–18% higher than control	[45]
Fucoidan derived from *Undaria pinnatifida* (Pectin)	Gibel carp, *Carassius auratus gibelio*; 5 g	30 g/kg diet	6 weeks 16–32% higher than control	[31]
*Enteromorpha prolifera* polysaccharide (Pectin)	Crucian carp, *Carassius auratus*; 51.24 g	20–40 g/kg diet	60 days 18–38% higher than control	[32]
Fermented wheat bran polysaccharide (Starch)	Common carp, *Cyprinus carpio*; 10 g	1–4 g/kg diet	8 weeks 14–26% higher than control	[4]
*Astragalus membranaceus* polysaccharide (Hemicellulose)	Crucian carp, *Carassius auratus*; 1.04 g	50–100 mg/kg diet	60 days 10–20% higher than control	[22]
Xylooligosaccharide (XOS) (Hemicellulose)	Grass carp, *Ctenopharyngodon Idella*; 3.05 g	0.05–0.2% of diet	8 weeks 16–18% higher than control	[46]
Pectin derived from orange peel (Pectin)	Common carp, *Cyprinus carpio*; 16.9 g	0.5–2% of diet	8 weeks 12–34% higher than control	[38]
Pectin derived from apple peel (Pectin)	Common carp, *Cyprinus carpio*; 16.89 g	0.5–2% of diet	8 weeks 38–53% higher than control	[39]
Non-starch polysaccharide (Hemicellulose)	Yellow River carp, *Cyprinus carpio*; 49.82 g	0.05–0.1% of diet	56 days 2–16% higher than control	[47]
Alginate oligosaccharide (Hemicellulose)	Grass carp, *Ctenopharyngodon*; 6.12 g	100–400 mg/kg diet	60 days 12–32% higher than control	[33]
*Ficus carica* polysaccharide (FCP) (Pectin)	Crucian carp, *Carassius auratus*; 40.16 g	0.01–0.4% of diet	21 days 43–107% higher than control	[23]
*Astragalus* polysaccharide (APS) (Hemicellulose)	Catla, *Catla catla*; 41.7 g	200–300 mg/kg diet	8 weeks 16–97% higher than control	[24]
*Taraxacum mongolicum* polysaccharide (Hemicellulose)	Jian carp, *Cyprinus carpio var Jian*; 5 g	0.5–2 g/kg diet	56 days 21–69% higher than control	[25]

## 4. Role of Polysaccharides and Innate Immunity Activation in Carp Farming

The gut microbiota is the microbial community colonizing the host digestive system [48]. Interaction between the host and gut microbiota will influence the host’s physiological, disease, and health status. The stability of the gut microbiota can be used as an indicator of the host’s health status. An imbalanced gut microbiota has been linked to multiple metabolic diseases. Short-chain fatty acids (SCFAs) are metabolites produced by gut microbiota that can pass through the digestive system and move into host cells. In addition, the SCFAs can interact with cells and influence the host’s immune response [49].

The role of polysaccharides as an immune system modulator in fish is well-documented. Polysaccharide enhances the fish immune system by promoting the growth of beneficial gut microbiota and eliminating pathogenic microorganisms in the host [50,51]. Polysaccharides activate the host defense cells and mediate the immune response [52], and the body eliminates the affected or infected cells. Furthermore, cytokines and chemokines generation will be promoted, thus activating the host immune system. Examples of medicinal herbs that could be used to derive polysaccharides are *Astragalus* spp., *Lycium barbarum*, and *Amorphophallus konjac*. Yuan et al. [53] and Shi et al. [54] agreed that *Astragalus* polysaccharide (APS) promotes the growth of beneficial gut microbiota in fish and modulates the immune system in carp farming. Nonetheless, the route of administration of APS differed in these studies. The APS was intraperitoneally injected in the study by Yuan et al. [53], while Shi et al. [54] proposed dietary administration of APS as a feed additive. Polysaccharide administration via intraperitoneal injection was labor-intensive, but the efficacy was evident within a shorter period than the feed additive route (56 days) (Table 2). Another medicinal herb polysaccharide that potentially acts as an immune modulator is *Lycium barbarum* polysaccharide (LBP). Zhou et al. [55] and Zhu et al. [56] reported that LBP served as a prebiotic that promoted the growth of beneficial gut microbiota, such as *Lactobacillus acidophilus* and *Bifidobacterium longum*. Likewise, *L. acidophilus* and *B. longum* in a host also gained benefits from the oxidized konjac glucomannan (KGM) and xylooligosaccharide (XOS) as prebiotics [57,58].

The β-glucan is a commercial polysaccharide naturally found in microorganisms and plants, reportedly enhancing the host’s immune system [59]. The roles of β-glucan in modulating the immune system of aquatic animals have been highlighted in previous studies [60,61,62]. For instance, β-glucan activated the immune system of carp in studies by Kuhlwein et al. [63], Falco et al. [64], and Pionnier et al. [65]. Furthermore, Kuhlwein et al. [63] suggested that incorporating β-glucan at 1–2% in carp for eight weeks was ideal. In contrast, Falco et al. [64] and Pionnier et al. [65] proposed a lower dose of 0.1% β-glucan for immune system enhancement in carp farming. In addition, most studies have agreed that oral administration of β-glucan is suitable for carp farming. Besides β-glucan, XOS could also boost carp’s immune system, as Zhang et al. reported [46]. Currently, β-glucan and XOS are the main inexpensive and abundantly available polysaccharides in the market. Therefore, these polysaccharides are cost-effective alternatives for modulating the carp’s immune system (Table 2).

**Table 2:** The role of polysaccharides in activating the innate immunity of carp.

Polysaccharides (Category) and Dose	Species and Weight	Indicator of Immunity	Duration	References
APS (Hemicellulose) 5–50 mg/kg fish	Common carp, *Cyprinus carpio*, 80 g	Gene expression	Intraperitoneal injection	[53]
*Coriolus versicolor* polysaccharide (CVP) (Pectin), 0.5–1 g/kg diet	Crucian carp, *Carassius auratus*, 58.3 g	Hematological and biochemical parameters	5 weeks	[66]
β-glucan (Starch) (MacroGard), 1–2% of diet	Mirror carp, *Cyprinus carpio*, 40 g	Hematological and blood biochemical parameters	8 weeks	[63]
β-glucan (MacroGard), 0.1% of diet	Common carp, *Cyprinus carpio*, 40 g	Gene expression	25 days	[64,65]
LBP, 0.5–1% of diet	Common carp, *Cyprinus carpio*, 150 g	Serum and blood biochemical parameters	60 days	[67]
Rare earth–chitosan chelate (Chitin), 0.8 g/kg diet	Gibel carp, *Carassius auratus gibelio*, 14.3 g	Blood biochemical parameters	12 weeks	[44]
*Hericium caput-medusae* polysaccharide (Pectin), 800–1200 mg/kg diet	Grass carp, *Ctenopharyngodon Idella*, 15.3 g	Blood biochemical parameters, Gene expression	2–3 weeks	[68]
*Radix rehmanniae preparata* polysaccharide (Inulin), 0.2% of diet	Common carp, *Cyprinus carpio*, 46 g	Gene expression	60 days	[45]
Pectin derived from orange peel (Pectin), 0.5–2% of diet	*Luciobarbus capito*, 16.9 g	Blood biochemical parameters	8 weeks	[38]
XOS, 0.1% of diet	Grass carp, *Ctenopharyngodon Idella*, 3.05 g	Blood biochemical parameters	8 weeks	[46]
APS (Hemicellulose), 1 g/kg diet	Grass carp, *Ctenopharyngodon Idella*, N.A	Blood biochemical parameters, Gene expression	56 days	[54]
Alginate oligosaccharide, 100–400 mg/kg diet	Grass carp, *Ctenopharyngodon idella*, 6.12 g	Blood biochemical parameters, Gene expression	60 days	[33]
Pectin derived from apple peel (Pectin), 0.5–2% of diet	Common carp, *Cyprinus carpio*, 16.89 g	Blood biochemical parameters	8 weeks	[39]
FCP, 0.4% of diet	Crucian carp, *Carassius auratus*, 40.16 g	Blood biochemical parameters	21 days	[23]
APS (Hemicellulose), 200 mg/kg diet	Catla, *Catla catla*, 41.7 g	Blood biochemical parameters, Gene expression	8 weeks	[24]
*Taraxacum mongolicum* polysaccharide (Hemicellulose), 1 g/kg diet	Jian carp, *Cyprinus carpio var Jian*, 5 g	Blood biochemical parameters, Gene expression	56 days	[25]

*Lycium barbarum* polysaccharide (LBP); *Astragalus* polysaccharide (APS); xylooligosaccharide (XOS); *Ficus carica* polysaccharide (FCP); not available (N.A).

## 5. Roles and Mode of Action of Polysaccharides and Disease Resistance Enhancement in Carp Farming

Various studies have revealed the potential of polysaccharides in enhancing carp disease resistance against *Aeromonas hydrophila* [69,70,71], while few have observed the disease resistance against viruses [72,73] and other bacteria such as *Edwardsiella tarda* [24] and *Staphylococcus aureus* [74]. Furthermore, the polysaccharide is commonly administrated via three different routes to enhance disease resistance in carp: (1) intraperitoneal injection, (2) oral gavage, and (3) supplemented feed. The intraperitoneal injection and oral gavage of polysaccharides are laborious but immediately effective compared to polysaccharide-supplemented feed, which requires several weeks to stimulate carp disease resistance. Tzianabos [75] reported that polysaccharide efficacy is principally influenced by the dose, administration route, and duration. Despite the slow action of polysaccharides as a feed additive, this method is widely applied due to the convenience of managing large-scale carp farming.

Polysaccharides derived from medicinal herbs, seaweed, bacteria, and commercial products, such as β-glucan, chitosan, XOS, and mannan oligosaccharide (MOS), could stimulate disease resistance in carp farming (Table 3). For example, *Coriolus versicolor* polysaccharide (CVP) at a dose of 1 g/kg diet stimulated carp resistance to *A. hydrophila* but demonstrated an adverse effect on the aquaculture species when included at higher doses. Moreover, high mortality was recorded when the carp were treated with polysaccharides with 2–4 g/kg diet [66]. Therefore, an appropriate dosage is crucial for incorporating polysaccharides in carp farming. In addition, β-glucan not only promoted host disease resistance against various microorganisms but also possessed a wound-healing property that was evident in common carp when the treatment was administered via bathing [76,77]. Furthermore, chitosan demonstrated antimicrobial activity and induced gibel carp resistance towards *A. hydrophila* infection when administered at a dose of 7500 mg/kg diet [42]. These polysaccharides are inexpensive and commercially available in the market, thus making them suitable for improving carp farming productivity.

## 6. The Role of Polysaccharides in Alleviating Abiotic Stress for Carp Farming

Abiotic factors are vital for fish growth and production in an aquaculture system [89]. Disruptions or fluctuations of these factors can affect the fish feeding behavior, resulting in sudden death or disease outbreak. Pollutants such as chemicals, herbicides, and pesticides in an aquaculture system are potential abiotic stressors to an aquaculture species. Examples of these abiotic stressors include carbon tetrachloride (CCl_4_), a chemical commonly used in the cleaning industry [90] and dioxin, an inorganic pollutant traced from the by-products of industrial and combustion processes [91]. Liu et al. [67] revealed that LBP provided hepatoprotection against CCl_4_ when given as a feed additive to the carp for 60 days. Meanwhile, a 4 h *Ganoderma lucidum* polysaccharide (0.1–0.6 mg/mL) treatment protected the CCl_4_-induced carp liver by suppressing the inflammatory immune response, inhibiting lipid peroxidation, and increasing antioxidant enzyme activity [92]. In addition, feeding Jian carp with feed containing *Glycyrrhiza glabra* polysaccharide (1.0 g/kg) for 60 days allowed the carp to withstand the toxicity of 2,3,7,8-tetrachlorodibenzo-*p*-dioxin (TCDD), one of the most toxic man-made substances [93]. To date, only polysaccharides derived from medicinal herbs are known to relieve the impacts of abiotic stress factors such as CCl_4_ and TCDD in carp farming. Nevertheless, the study of polysaccharides to alleviate abiotic stress in carp farming remains limited. One possible mode of action of these polysaccharides is enhancing the fish’s immune system. According to Chaplin [50], an immune system booster can help the host to eliminate toxins. Therefore, polysaccharides can strengthen the carp’s immune system and protect it from harmful toxins.

## 7. Combination of Polysaccharides and Other Prophylactic Agents to Improve Carp Farming

Combining polysaccharides with other prophylactic agents such as probiotics amplified the performance of this molecule compared to a single polysaccharide and served as a prebiotic to the probiotics [94,95]. Thus, symbiotic application, or a combination of prebiotic and probiotic dietary supplements that yields synergistic effects, has gained the attention of researchers worldwide [96]. For instance, a diet supplemented with a combination of galactooligosaccharide (GOS) and *Pediococcus acidilactici* exhibited a pronounced effect on several mucosal or serum immune parameters in common carp [97]. Furthermore, polysaccharide combinations such as β-glucan and MOS with *Lactobacillus casei* [98], and XOS with *Bacillus subtilis* [99], enhanced the growth performance, immune response, and disease resistance of carp against *A. hydrophila.* (Table 4). Moreover, combining dietary polysaccharides and prophylactic agents in carp farming improved the overall health status of the fish, including the growth performance, immune response, disease resistance, and survival rate, by regulating and balancing the gut microbiota and the expression of inflammation-related genes [100].

## 8. Conclusions and Recommendations

Carp farming is an important aquaculture activity in many countries. However, this aquaculture activity is constantly hampered by disease outbreaks. The application of non-antibiotic prophylactic agents in aquaculture species health management is rising due to the concern about antibiotic residues in aquaculture products. Nevertheless, antibiotics remain the fast-action solution in aquaculture to effectively overcome diseases. Conversely, polysaccharides require a longer period as a prophylactic agent. Polysaccharides derived from medicinal herbs, seaweeds, and agricultural wastes and obtained from commercial products are popular prophylactic agents in carp farming. In this review, it was evident that polysaccharides are promising in promoting growth performance, enhancing the immune system, stimulating disease resistance, and relieving abiotic stress factors in carp farming. Numerous studies have demonstrated that administrating polysaccharides as a feed supplement in carp farming is preferred compared to intraperitoneal injection and oral gavage. Polysaccharide is an economical feed supplement on a large scale despite the longer efficacy period. Nonetheless, determining the proper polysaccharide dosage is crucial to prevent overdosing and undesirable impacts on carp growth performance. A change in the approach of future studies is necessary to identify the molecular and cellular pathways that regulate the immune responses of carp towards different pathogens and abiotic stressors; thus, we might fully understand the effects of polysaccharides in combination with other agents in carp farming. Despite the synergistic effects demonstrated by the combination of polysaccharides and other prophylactic agents, it is essential to perform an in-depth evaluation of the long-term efficacy and safety of these compounds for sustainable carp farming.

## Figures and Tables

**Table 3:** Polysaccharides in enhancing diseases resistance of carp farming.

Polysaccharides (Category)	Species and Weight	Dose and Duration	Pathogen and Dose	Reference
Lentinan, *Schizophyllan, scleroglucan* (Starch)	Common carp, *Cyprinus carpio*; 25–30 g	5 mg/kg fish (intraperitoneal injection) Thrice—1st, 4th, and 7th day before exposure to pathogen	*A. hydrophila*, 10^7^ CFU	[69]
Sodium alginates, polysaccharide derived from *Undaria pinnatifida* (Pectin)	Grass carp, *Ctenopharyngodon Idella*; 25–30 g	30 mg/kg fish (intraperitoneal injection) Twice—3rd and 6th day before exposure to pathogen	*E. tarda*, 10^7^ CFU	[78]
Polysaccharide extracted from barley, krestin, scleroglucan, zymosan	Common carp, *Cyprinus carpio*; 24 g	10 mg/kg fish (intraperitoneal injection) Twice—2 days interval	*A. hydrophila, E. tarda*, 10^7^ CFU	[70]
β-glucan (Starch)	Grass carp, *Ctenopharyngodon Idella*; 8.5 g	10 mg/kg fish (intraperitoneal injection) 15 days	Grass carp hemorrhage virus (GCHV), 5 LD_50_/mL	[72]
Lipopolysaccharide (LPS) (Lipopolysaccharide) from virulent *A. hydrophila*	Common carp, *Cyprinus carpio*; 25–30 g	10–100 µg/fish (intraperitoneal injection); Day 1, 7, and 14 15–150 µg/mL (bathing); Day 1, 7 and 14	*A. hydrophila*, 2.11 × 10^7^ CFU	[79]
β-glucan (Starch) (MacroGard)	Common carp, *Cyprinus carpio*; 78.4 g	6 mg/kg fish; 14 days	*A. salmonicida*, 4 × 10^8^ CFU	[80]
CVP (Pectin)	Crucian carp, *Carassius auratus*; 58.3 g	1 g/kg diet; 56 days	*A. hydrophila*, 10^6^ CFU	[66]
MOS (Hemicellulose)	Grass carp, *Ctenopharyngodon Idella*; 16.19 g	240–480 mg/kg diet; 10 weeks	*A. hydrophila*, 10^8^ CFU	[81]
β-glucan (Starch)	Common carp, *Cyprinus carpio*; 78.4 g	6 mg/kg fish; 14 days	*A. salmonicida*, 10^8^ CFU	[82]
FCP (Pectin)	Grass carp, *Ctenopharyngodon Idella*; 80 g	0.5–1%; 3 weeks	*Flavobacterium columnare,* 3.6 × 10^7^ CFU	[83]
*Ficus carica*, *Radix isatidis*, *Schisandra chinensis* polysaccharide (Pectin)	Crucian carp, *Carassius auratus*; 50 g	500 mg/kg diet; 21 days	*A. hydrophila*, 6 × 10^7^ CFU	[84]
*Padina gymnospora* polysaccharide (Pectin)	Common carp, *Cyprinus carpio*; 100 g	0.01, 0.1, 1% of fish diet; 1–3 weeks	*A. hydrophila, E. tarda*, 5 × 10^7^ CFU	[85]
*Hericium caput-medusae* polysaccharide (Hemicellulose)	Grass carp, *Ctenopharyngodon Idella*; 15.3 g	800 mg/kg diet; 3 weeks	*A. hydrophila*, 2 × 10^6^ CFU	[68]
*Porphyra yezoensis* Polysaccharide (Pectin)	Grass carp, *Ctenopharyngodon Idella*; 6.12 g	3 g/kg diet; 60 days	*A. hydrophila*, 1.5 × 10^5^ CFU	[30]
Polysaccharides derived from honeysuckle flowers (Hemicellulose)	Common carp, *Cyprinus carpio*; 35.23 g	250–1000 µg/mL (oral gavage); 5 days	*A. hydrophila*, 10^6^ CFU	[71]
*Radix rehmanniae preparata*	*Luciobarbus capito*; 46.2 g	0.2% of diet; 60 days	*A. hydrophila*, 2 × 10^7^ CFU	[45]
Chitosan nanoparticle (Chitin)	Silver carp, *Hypophthalmichthys molitrix*; 65 g	5 g/kg diet; 60 days	*S. aureus*, 10^7^ CFU	[74]
XOS (Hemicellulose)	Grass carp, *Ctenopharyngodon Idella*; 3.05 g	0.1% of diet; 8 weeks	*A. hydrophila*, 5.5 × 10^7^ CFU	[46]
*Enteromorpha prolifera* polysaccharide (Pectin)	Crucian carp, *Carassius auratus*; 51.24 g	20–80 g/kg diet; 60 days	*A. hydrophila*, 1.5 × 10^5^ CFU	[32]
*Astragalus membranaceus* polysaccharide (Hemicellulose)	Crucian carp, *Carassius auratus*; 1.04 g	100 mg/kg diet; 60 days	*A. hydrophila*, 10^7^ CFU	[22]
MOS (Hemicellulose)	Grass carp, *Ctenopharyngodon Idella*; 215.85 g	538.5–585.8 mg/kg diet; 60 days	*A. hydrophila*, 10^7^ CFU	[86]
Exopolysaccharide from *Lactococcus lactis* Z-2 (Lipopolysaccharide)	Common carp, *Cyprinus carpio*; 47.66 g	250–1000 µg/mL (oral gavage); 7 days	*A. hydrophila*, 5 × 10^6^ CFU	[87]
Alginate oligosaccharide (Hemicellulose)	Crucian carp, *Carassius auratus*; 6.12 g	100–400 mg/kg diet; 60 days	*A. hydrophila*, 1.5 × 10^5^ CFU	[33]
APS (Hemicellulose)	Grass carp, *Ctenopharyngodon Idella*; N.A	1 g/kg diet; 56 days	*A. hydrophila*, 10^6^ CFU	[54]
*Agaricus bisporus* polysaccharides (Hemicellulose)	Grass carp, *Ctenopharyngodon Idella*; 148.5 g	1 mg/kg diet; 60 days	*A. hydrophila*, 1.7 × 10^6^ CFU	[88]
APS (Hemicellulose)	Crucian carp, *Carassius auratus*; 30.5 g	2 g/kg diet; 56 days	Spring viremia of carp virus (SVCV), 10^7^ TCID_50_/100 µL	[73]
FCP (Pectin)	Crucian carp, *Carassius auratus*; 40.16 g	0.4% of diet; 21 days	*A. hydrophila*, 6 × 10^7^ CFU	[23]
APS (Hemicellulose)	Catla, *Catla catla*; 23.7–41.7 g	200–300 mg/kg diet; 8 weeks	*E. tarda*, 10^6^ CFU	[24]

*Lycium barbarum* polysaccharide (LBP); *Astragalus* polysaccharide (APS); xylooligosaccharide (XOS); *Ficus carica* polysaccharide (FCP); *Coriolus versicolor* polysaccharide (CVP); mannan oligosaccharide (MOS); colony-forming unit (CFU); not available (N.A).

**Table 4:** Combination of polysaccharide and prophylactic agent to improve carp farming.

Polysaccharides (Category) (Dose)	Species and Weight/Stage	Prophylactic Agent (Dose)	Duration	Effects	Reference
GOS (Hemicellulose) (10 g/kg diet)	Common carp, *Cyprinus carpio*, juvenile	*Pediococcus acidilactici* (0.9 × 10^7^ CFU/kg diet)	8 weeks	Enhanced serum and mucosal immune response	[97]
β-glucan (Starch) and MOS (Hemicellulose) (1% of diet)	Common carp, *Cyprinus carpio*, 65 g	*Lactobacillus casei* (5 × 10^7^ CFU/kg diet)	60 days	Enhanced immune system and disease resistance to *A. hydrophila*	[98]
XOS (Hemicellulose) (0.1% of diet)	Crucian carp, *Carassius auratus*, 9.77 g	*Bacillus subtilis* (1 × 10^8^ CFU/g diet)	8 weeks	Improved growth performance, survival rate, immunity, and disease resistance to *A. hydrophila*	[99]

Xylooligosaccharide (XOS); galactooligosaccharide (GOS); mannan oligosaccharide (MOS).

## Data Availability

Not applicable.
